# Rediscovery of four narrow endemic *Didymocarpus* species (Gesneriaceae) from Mizoram, India, with revised species descriptions and lectotypifications

**DOI:** 10.3897/phytokeys.148.49772

**Published:** 2020-05-20

**Authors:** Naibi Shrungeshwara Prasanna, Vinita Gowda

**Affiliations:** 1 Tropical Ecology and Evolution (TrEE) Lab, Department of Biological Sciences, Indian Institute of Science Education and Research, Bhopal, 462066, India Department of Biological Sciences, Indian Institute of Science Education and Research Bhopal India

**Keywords:** C.E.C. Fischer, gesneriads, Lushai hills, nomenclature, Northeast India, pair-flowered cyme, taxonomy

## Abstract

Here we report the rediscovery of four endemic gesneriads from the state of Mizoram, India, after a span of 86 to 90 years since their last collection. The four species belong to the genus *Didymocarpus* Wall. and they are: *D. adenocarpus*, *D. lineicapsa*, *D. parryorum*, and *D. wengeri*. We present revised morphological descriptions, photographs, and designate lectotypes for *D. parryorum* and *D. wengeri*. During our study we came across several discrepancies between morphological characters assigned to these four species in the protologue and morphological characters present (or absent) in the type specimens and in plants recollected by us. We list these discrepancies in a section titled ‘amendments to protologue’. Based on the high endemicity and critical conservation status of all the four rediscovered species, we suggest that floristic studies along with large-scale biogeographic studies should be prioritized in the Indo-Burmese region.

## Introduction

The genus *Didymocarpus* Wall. was recently redefined by Weber and Burtt (1998) and it now consists of approximately 100 species that are distributed in India, Nepal, Bhutan, southern China, Myanmar, Thailand, Vietnam, Laos, Cambodia, Peninsular Malaysia, and Sumatra (Möller 2019). Phylogenetic studies by Palee et al. (2006) suggested the geographic origin of the genus to be Malay Peninsula, although the northeast region of India along with southern China accounts for more than half of known *Didymocarpus* species (Möller et al. 2017; Möller 2019). India is known to have about 25 species of *Didymocarpus* most of which are narrow endemics, restricted to relatively unexplored areas of Northeast India (Prasanna et al. 2020). Within India, they are mainly distributed in the Indo-Burmese and Eastern Himalayan region with one species extending into the Western Himalayas (Möller et al. 2017; Roy 2017).

**Figure 1.**
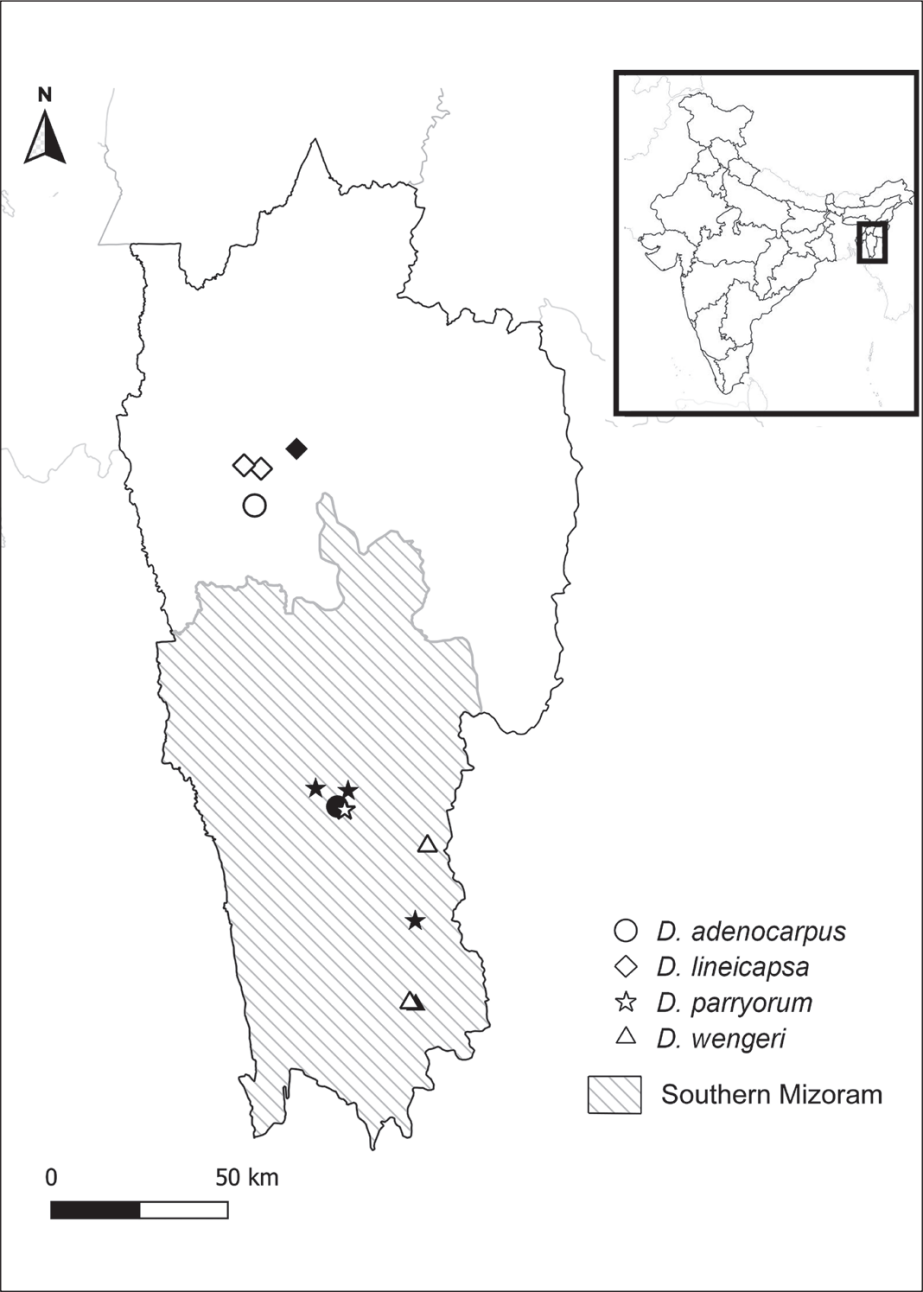
Map showing distribution records of the four rediscovered species of *Didymocarpus* Wall. (Gesneriaceae) in Mizoram, India. Solid symbols indicate historical collection sites and open symbols indicate extant populations. Hatched area represents southern Mizoram which is referred to as ‘South Lushai hills’ in the protologues and historical collections.

Mizoram, formerly known as Lushai hills, is a small state in the northeast region of India and was part of the state of Assam until 1972. It is bordered to the west by Bangladesh and to its east and south by Myanmar, and it is part of the Indo-Burmese biogeographic region. Two of the earliest colonial-era plant collectors who can be credited with collecting gesneriads in this region are Mrs Anne Parry (commonly cited as Mrs N.E. Parry, *pers. comm.* H. Noltie) and Rev. W.J.L. Wenger. Parry is known to have accompanied her husband, a British officer, who was appointed as the Superintendent of Lushai hills, Mizoram (then Assam) from 1924–1928 (Parry 1932). Wenger was a Baptist missionary who was sent to work at the Baptist church of Lunglei, Mizoram from 1922 to 1933 (Lalzama 1990). During their stay in Mizoram, both Parry and Wenger independently made extensive plant collections within the northeast region of India and they regularly sent their collections to Kew, where C. E. C. Fischer identified and described these species and published floristic studies (Fischer 1928a–c, 1938). Here we report rediscoveries of four of these species which were first collected by either Parry or Wenger and later described by Fischer. All four gesneriads reported here have not been collected since either Parry or Wenger collected them in the early 1900s, despite recent revisionary, taxonomic, or floristic studies carried out in the Northeast of India (Sinha 2012; Sinha and Datta 2016; Roy 2017). Thus, our rediscovery of the four species is clocked at approximately 86 to 90 years since their last collection. Our observations suggest that all four species are narrow endemics within the state of Mizoram, India, which was also noted by earlier botanists like Wenger (Fischer 1928a) in the type description (Fig. 1).

The rediscovery of the four *Didymocarpus* species is an outcome of our concerted effort to collect gesneriads from the Northeast of India for our ongoing revision of the genus using a molecular phylogenetic approach. Although there have been several revisionary studies of *Didymocarpus* from India (Sinha 2012; Sinha and Datta 2016; Roy 2017), these studies either omitted to list a species (e.g., *D. lineicapsa*) rediscovered by us or we found errors in their taxonomic descriptions. We therefore utilized freshly collected material which allowed us to make an in-depth exploration of morphological characters that are taxonomically important but may be difficult to study in herbarium specimens. We have made several amendments to the current taxonomic descriptions of the four rediscovered species and these are given in a separate section titled ‘amendments to protologue’ (see materials and methods below). Finally, our description and taxonomic study of *D. parryorum* C.E.C.Fisch. and *D. wengeri* C.E.C.Fisch. also resulted in our discovery that Fischer did not assign unambiguous types to both these taxa. We therefore also assign lectotypes for both these species here.

## Materials and methods

Field expeditions were carried out throughout the monsoon season (July to September), when the plants are known to flower, in the years 2017 and 2018. Type localities of all *Didymocarpus* species in Mizoram and surrounding areas, including neighboring states, were visited. Due to logistic and financial constraints, we were unable to visit the field sites in the dry season and therefore the taxonomic descriptions listed here are based on rainy season forms only, although some species of *Didymocarpus* have been known to have morphologically distinct seasonal forms (Weber and Burtt 1998; Nangngam and Maxwell 2013). We carried out extensive metadata collection for each species which included scoring of morphological, phenological, reproductive and ecological characters such as flower opening time, pollinator visitation, and fruit set. Morphological measurements of five to six freshly dissected samples of each species were taken using both a ruler and a digital calliper. Plant materials used in this study include herbarium vouchers, spirit samples, and leaf tissues in silica for ongoing molecular phylogenetic studies. All herbarium vouchers collected by us are deposited at BHPL and duplicates will be deposited at ASSAM.

For taxonomic and nomenclatural work, we studied relevant *Didymocarpus* protologues along with following research materials: Kanjilal et al. 1939; Sinha 2012; Sinha and Datta 2016; Roy 2017. Herbarium collections including type specimens were consulted at ARUN, ASSAM, BHPL, CAL, E, K and BM and in online databases (Chinese Virtual Herbarium: http://www.cvh.ac.cn/en; Global Plants: https://plants.jstor.org/; Kew Herbarium Catalogue: http://apps.kew.org/herbcat/; Muséum National d’Histoire Naturelle: https://science.mnhn.fr/; Smithsonian Institution: https://www.si.edu/; The Linnaean Collections: http://linnean-online.org/; see Appendix 1). We evaluated the conservation status for the four rediscovered species according to the latest International Union for Conservation of Nature guidelines (IUCN 2019) using species distribution ranges and size of the populations we encountered during fieldwork.

In this study, we have modified the terms that describe bracteoles and glands in all the four species and we provide rationale wherever a different term from the protologue has been used. Within Gesneriaceae, many authors including Fischer (1928a–c, Spare and Fischer 1929) have used the term ‘bracts’ to indicate modified leaves present in the inflorescence. However, in pair-flowered cymes of *Didymocarpus*, the morphology of the bracts present at the base of the primary fork may vary from those subtending the subsequent forks. To bring taxonomic clarity, we find it necessary to differentiate between these two structures and here, we use the term ‘primary bracteoles’ to indicate the modified leaves subtending the primary forks and ‘secondary bracteoles’ to indicate all the bract-like structures subtending the subsequent forks within the inflorescence.

We have added a new section ‘amendments to protologue’ which is an important part of the updated taxonomic description for all the four rediscovered species. This section has been added because we found that the morphological description of all four rediscovered species of *Didymocarpus* did not match those given in their respective protologues. Listing these discrepancies in a separate section allows us to avoid ambiguities in the description and therefore avoid future taxonomic confusions. In this section, we list all morphological differences we have noted between the protologue, type specimen and fresh specimens of the same species. Descriptive discrepancies may have resulted because Fischer wrote his taxonomic descriptions only from a limited number of herbarium material shipped to him by Parry and/or Wenger from Northeast India. Thus, the quality of the specimens may have resulted in ambiguous or erroneous descriptions (e.g., refer to discrepancy in stem color of *D. adenocarpus* and indumentum on corolla in *D. lineicapsa*).

## Results

### Didymocarpus adenocarpus

Taxon classificationPlantaeLamialesGesneriaceae

C.E.C.Fisch., Bull. Misc. Inform. Kew 1929: 253. 1929.

DAADBEB8-A639-5C29-94A7-ABFD806D3BAF

**Figure 2.**
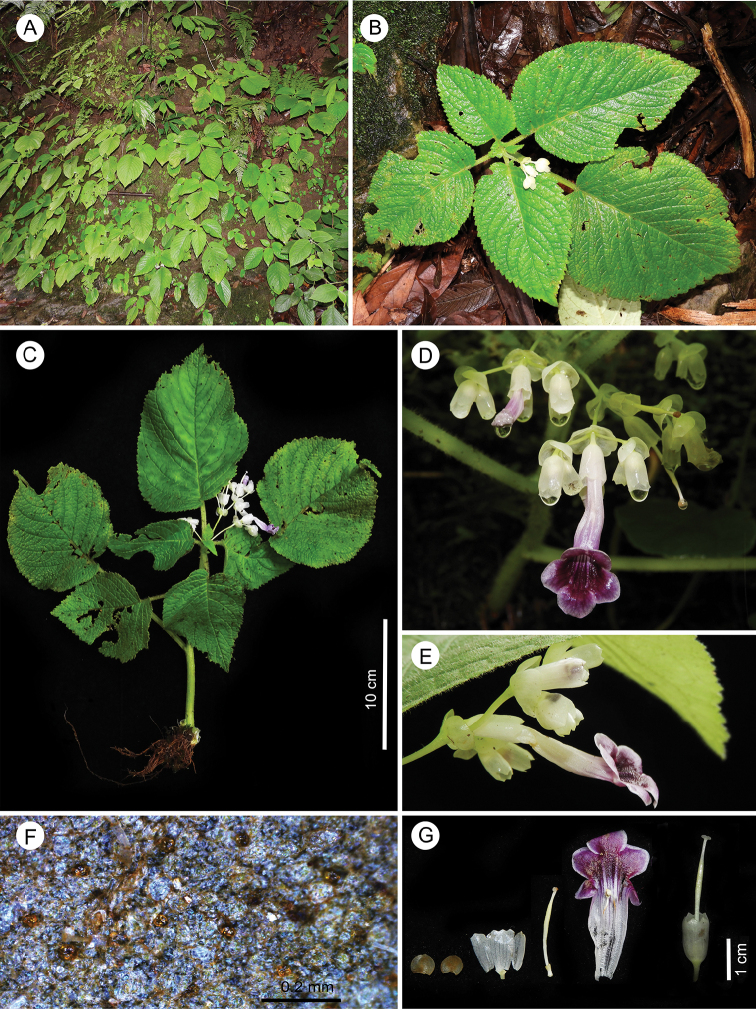
*Didymocarpus adenocarpus* C.E.C.Fisch. **A** habitat **B** habit **C** complete plant with inflorescence **D** pair-flowered cyme **E** flower, side view **F** glands on abaxial surface of leaves **G** floral dissection (from left to right): primary bracteole, secondary bracteole, sepal, gynoecium, open floral tube showing fused anthers, gynoecium surrounded by persistent calyx. Photographs by NSP.

Fig. 2A–G , Suppl. material 1: Fig. S1A, B

#### Holotype.

India. Assam (= Mizoram): Southern Lushai Hills, 4500 ft., Sept. 1928, Rev. W.J.L. Wenger 239, K (K000820546!).

#### Revised description.

Terrestrial or epilithic herbs, up to 35 cm tall. Stem 16 × 6 mm, terete, light green, sparsely pubescent with 4–10 celled eglandular hairs. Leaves 2–6 pairs, opposite and anisophyllous, decussate, terminal pair smaller in size, membranous, exstipulate; petioles 1–8 cm long, pubescent with multicellular eglandular hairs as on stem; lamina 9–15 × 5–8 cm, oblong to orbicular, lamina separated unequally by midrib, base cordate to obliquely cordate, apex acute to acuminate, margin coarsely crenate-dentate; dorsal surface green, sparsely pubescent with multicellular eglandular hairs; ventral surface pale green, densely pubescent along veins but sparsely pubescent otherwise; densely dotted with minute globose, pale-brown glistening pigment glands (in dried specimen); midrib with 6–10 lateral veins on either side, sunken above, raised below, secondary veins more prominent. Inflorescence 1 to 4, pedunculate, axillary, pair-flowered cymes (many-flowered), usually arising only from the axils of the 1–2 uppermost pairs of leaves, cyme with up to 20 flowers; primary bracteoles present, 4 × 7 mm, opposite, suborbicular, apex mucronate, glabrous, translucent white, veins visible when dried; secondary bracteoles (within the cyme) present at each dichotomous fork, 4 × 6 mm, suborbicular, apex mucronate glabrous, whitish, veins visible when dried. Inflorescence usually hidden below the leaves, pendent; peduncle 3–4 cm long, light green, lower part sparsely pubescent with multicellular eglandular hairs, upper part glabrous; pedicel ca. 5 mm long, slender, glabrous; Calyx 0.8–1 mm long, fused, narrowly funnel shaped, with 5–9 short, broadly triangular teeth with a visible vein running into each, glabrous, whitish, translucent; Corolla 2.8–3.5 cm × 0.5–0.8 cm, tubular with a slight bend, infundibuliform towards mouth; tube whitish at base, purple towards lobes; corolla bi-lipped, total 5 lobes, 0.5 × 0.5 cm, suborbicular, glabrous, purplish with whitish outer edge, the 3 lower lobes larger than the 2 upper lobes, ventral part of the corolla tube and lobes striated. Stamens 2, inserted at 1/3^rd^ of the length of the tube from the mouth of the corolla, anthers dorsifixed, coherent by adaxial surfaces; filaments 1–1.2 cm, glabrous, whitish; staminodes 2 or 3, inserted lower than the stamens, tip bifurcated, the third when present below the others and much shorter. Disc up to 2 mm long, tubular with undulating upper margin, glabrous, persistent. Gynoecium 2.1– 2.2 cm, ovary linear, slightly widened upwards, glabrous, covered with globose yellow glands; stigma peltate, glabrous. Capsule linear, brown, 3–3.5 cm long, dotted with glistening yellowish glands. Seeds very minute, pale-reddish-brown, fusiform, acute at both ends.

#### Amendments to protologue.

Upon examining fresh specimens (Fig. 2 and Suppl. material 1: Fig. S1A, B), we noted that the stem color is light green (brown in protologue), corolla tube is whitish at base, purple towards lobes (white tinged with pink in protologue), and bracteoles are translucent white (reddish brown in protologue). Leaf apex is acute to acuminate (acute or abruptly acutely cuspidate in protologue) and inflorescence is typical pair-flowered cyme (central 1-flower and trichotomous branching in protologue). We found stigma to be glabrous (pubescent in protologue).

#### Note.

*D. adenocarpus* is similar to *D. purpureobracteatus* W.W.Sm. but differs from it in having slightly cordate leaves (rounded or oblique in *D. purpureobracteatus*), sparsely pubescent peduncle (glabrous in *D. purpureobracteatus*), and glabrous pistil (sparsely puberulent in *D. purpureobracteatus*).

#### Distribution.

Historically, *D. adenocarpus* is known from southern Mizoram. However, in this study we located one extant population at Reiek Tlang in Mamit district of northern Mizoram (specimen numbers: VG2018MZ2589, VG2018MZ2590, VG2018MZ2592).

#### Habitat.

Grows on moist loamy banks in partially shaded areas of tropical wet evergreen forests.

#### Phenology.

Flowering in August to September, fruiting in September to December.

#### Ecology.

We observed that *D. adenocarpus* has a tubular calyx which can retain and immerse the buds in water (see Suppl. material 1: Fig. S1B). In other gesneriads such as *Aeschynanthus* and *Chrysothemis*, a similar character was referred to as watery calyces, and was suggested as a mechanism to reduce florivory by insects (Carlson and Harms 2007).

#### Conservation status and preliminary IUCN assessment.

*D. adenocarpus* is known from only four specimens collected from southern Mizoram, India. To the best of our knowledge there have been no further collections of *D. adenocarpus* until this study, which brings the time until its current rediscovery up to 87 years. We surveyed multiple potential locations in Mizoram and we could not locate any population in southern Mizoram. The extant population is limited to an area of about 15 km^2^ in Reiek Tlang hills, Mamit district, which is in northern Mizoram. Although it is a community protected forest, with limited anthropogenic disturbance, the population has only 300 mature individuals. Therefore, based on the criterion C2a(i) of IUCN guidelines (IUCN 2019), we propose that the species should be considered as endangered (EN).

### Didymocarpus lineicapsa

Taxon classificationPlantaeLamialesGesneriaceae

(C.E.C.Fisch.) B.L.Burtt, Notes Roy. Bot. Gard. Edinburgh 21(4): 187. 1954.

29198D6B-730C-585A-B78C-6253164F951A

**Figure 3.**
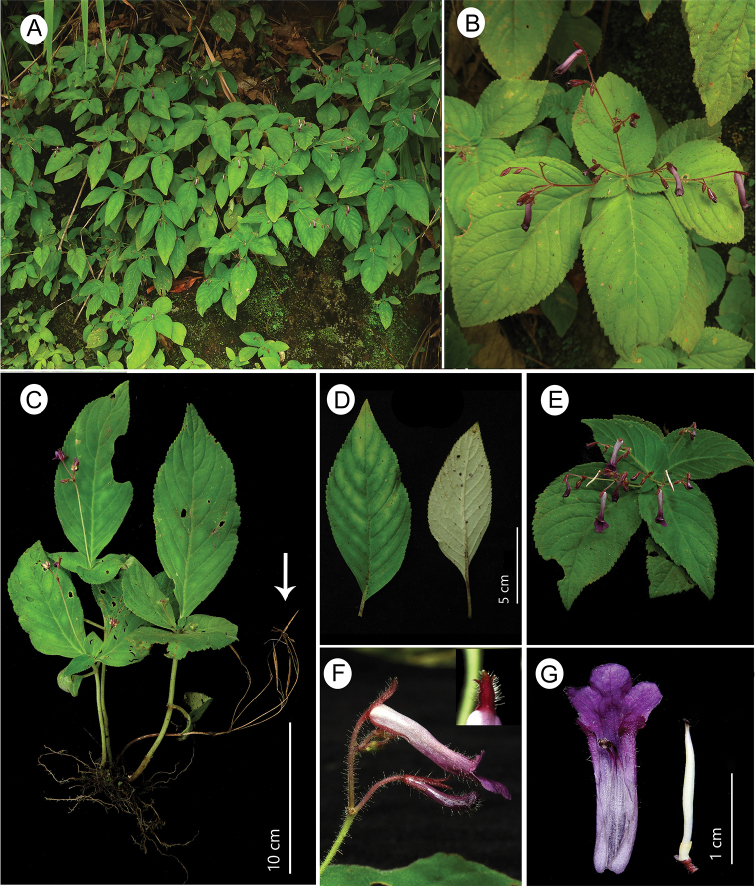
*Didymocarpus lineicapsa* (C.E.C.Fisch.) B.L.Burtt **A** habitat **B** habit **C** complete plant with emerging inflorescence. Old stem with dehisced capsules from previous season indicated by arrow **D** leaf adaxial and abaxial surface **E** inflorescence **F** flower (inset - tridentate calyx lobe) **G** floral dissection from left to right: open floral tube showing fused anthers, gynoecium with disc and ovary. Photographs by NSP.

Fig. 3 , Suppl. material 1: Fig. S1D

#### Basionym.

*Trisepalum lineicapsa* C.E.C.Fisch., Bull. Misc. Inform. Kew 1928 (7): 276 (1928).

#### Holotype.

India. Assam (= Mizoram): Lushai Hills, Aijal (= Aizawl), 1225 m, September 1927, Mrs N.E. Parry No.79, K (K000820539!).

#### Revised description.

Terrestrial or epilithic herbs, to 15 cm tall, 1 to 4 stems arising from the same rhizome. Stems 3 to 15 cm long, 2–4 mm wide at base, erect, dark green, terete, densely tomentose with 3 to 4 celled eglandular hairs and sparsely interspersed globular, yellow pigment glands. Leaves 4–6 pairs, opposite and anisophyllous, decussate, often whorled at the top; petioles up to 2.7 cm long, terete, densely tomentose as on stem, sparsely covered with globular, yellow pigment glands; lamina 3–10 cm × 1.5–3.5 cm, lanceolate to narrowly elliptic, lamina separated unequally by midrib, base oblique, apex acute; margin dentate, often entire towards the base, dorsal surface dark green, densely strigose with short eglandular hairs, ventral surface light green, strigose with yellow-glandular (colour as observed in dried specimen) and eglandular hairs, hairs more dense along the veins; midrib with 8–10 secondary veins on each side, sunken above, raised below. Inflorescence 1 to 4, axillary, spreading from upper leaves forming the whorl, erect, pair-flowered cymes (many-flowered), usually arising only from the axils of the 1–2 uppermost pairs of leaves; peduncle 1.5–6 cm long, up to 5 mm thickness (slender), sparsely covered with multicellular glandular and eglandular hairs; pedicel up to 2 cm long, pale pink, covered with multicellular glandular and eglandular hairs; bracteoles absent. Calyx 5–6.5 mm long, maroon coloured, tripartite; two segments up to 0.5 mm wide, linear-lanceolate, tip acute, free to base, held ventrally along the lower side of the corolla tube; third segment tridentate, up to 1.2 mm wide, held dorsal to the corolla tube, central tooth wider than the two lateral teeth; dorsal surface glandular-pubescent; ventral surface glabrous. Calyx not persistent. Corolla 1.5–1.8 cm long, ca.2.2 mm wide, tubular, light purple at base but dark purple towards throat and lobes. Corolla tube usually held perpendicular to the pedicel; corolla tube glabrous at base but with multicellular glandular hairs below the lobes, hairs sometimes present also on lower part of the lobes, corolla tube glabrous on the inside; corolla bi-lipped, total 5 lobes; upper lobes 2, 1.6 × 3.1 mm, apices rounded; lower lobes 3, 6.5–7.5 × 3.5–4.5 mm, spreading at right angles to the upper lobes, middle lobe apex rounded, lateral lobes apices obtuse. Stamens 2, filament inserted at about 1/3^rd^ of the length of the corolla tube; filaments 5–6 mm, glabrous, filament dark purple near the anthers, anthers dorsifixed, coherent by adaxial surfaces, glabrous; staminodes absent. Disc up to 2 mm, tubular, yellowish, glabrous, upper margin undulate, persistent. Gynoecium 10–11 mm, ovary white, linear, indistinct from stipe, glabrous; style ca. 2 mm glabrous; stigma dark purple, capitate. Capsule 1.5–2.5 cm long, linear/straight, glabrous, longitudinal dehiscence. Seeds data not available.

#### Amendments to protologue.

The protologue by Fischer indicates that *D. lineicapsa* has bracts at each inflorescence fork (“*bracteae ad furcas*”). However, we observed that the holotype and other subsequent collections by Parry, Wenger as well as our own collections (Fig. 3), do not have any bracts or bracteoles within the inflorescence. The protologue also mentions that *D. lineicapsa* has a glabrous corolla tube, however all specimens including the type specimen have been found to be sparsely covered with multicellular, glandular hairs towards the lobes.

#### Note.

*D. lineicapsa* is similar to *D. graciliflorus* R.W.MacGregor & W.W.Sm. in its vegetative morphology but differs due to the absence of bracteoles (ovate bracteoles present in *D. graciliflorus*) and linear-lanceolate, tripartite calyx lobes (oblong 5-partite lobes in *D. graciliflorus*).

#### Distribution.

The type locality of *D. lineicapsa* is near Aizawl in northern Mizoram and subsequent collections are known from throughout the state. In our expeditions, we could not locate any populations in its type locality or historical collection sites. However, we found three scattered populations in Mamit district of northern Mizoram which is at least 40 km away from its type locality (specimen numbers: VG2018MZ2581, VG2018MZ2584, VG2018MZ2585, VG2018MZ2596).

#### Habitat.

These plants grow on steep clayey banks along the roads in partially shaded, tropical wet evergreen forests.

#### Phenology.

Flowering in August to September, fruiting in October to January.

#### Conservation status and preliminary IUCN assessment.

*D. lineicapsa* is known from only seven specimens collected from Mizoram, India, and it has not been recollected for the past 89 years. We carried out collection expeditions in the years 2017 and 2018 to the type location (Aizawl, Mizoram) as well as other historical collection sites (Fig. 1). All of the historical locations have undergone dramatic urbanization in the last eight decades and we could not find any population of *D. lineicapsa* in any of these sites. Instead, we found only three disjunct populations of *D. lineicapsa* with a total of less than 1000 individuals, in Mamit district, Mizoram. All the extant populations are located in rapidly degrading, fragmented forests that do not fall under federally protected areas, and therefore we propose the conservation status of this species as vulnerable (VU) following the criteria D2 of IUCN guidelines (IUCN 2019).

### Didymocarpus parryorum

Taxon classificationPlantaeLamialesGesneriaceae

C.E.C.Fisch., Bull. Misc. Inform. Kew. 1928(4): 142. 1928.

276A9F04-5A38-5811-B2A9-6465D47C0D08

**Figure 4.**
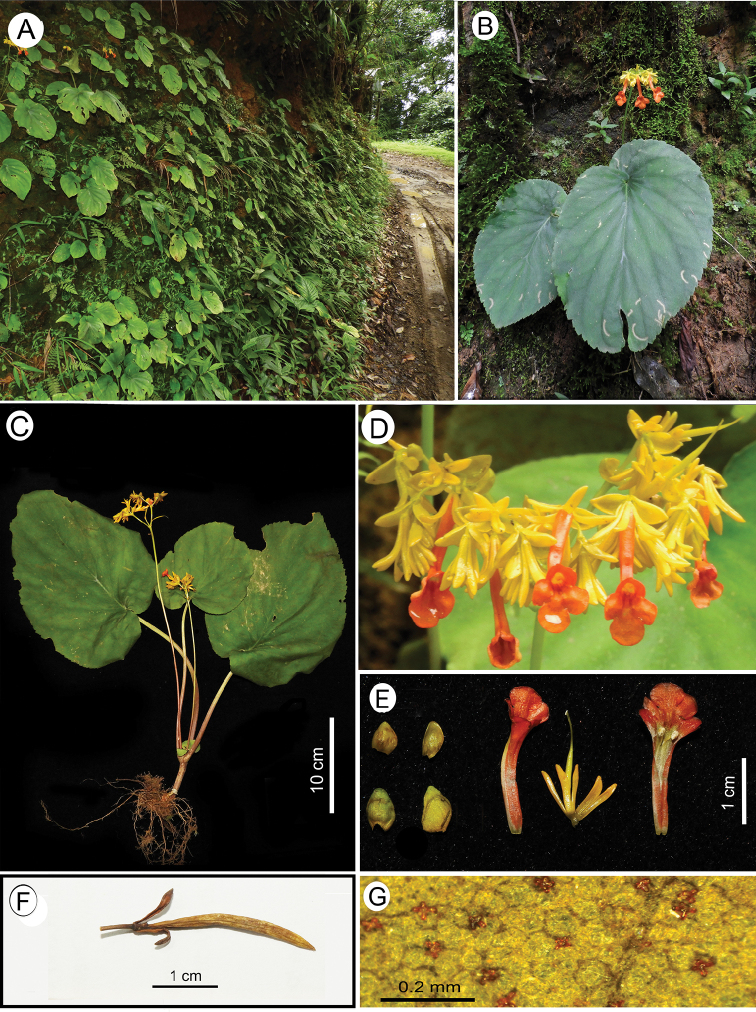
*Didymocarpus parryorum* C.E.C. Fisch. **A** habitat **B** habit **C** complete plant with inflorescence **D** inflorescence **E** floral dissection from left to right: primary bracteoles (above), secondary bracteoles (below), corolla tube, calyx with gynoecium, open floral tube with anther (fusion of anther lost in dissection of flower) **F** mature fruit **G** glands on the lower surface of leaves. Photographs by NSP.

Fig. 4 , Suppl. material 1: Fig. S1C, E

#### Lectotype

**(designated here).** India. Assam (= Mizoram), Lunglei district, Lushai hills at Sairep, 5000 ft., July 1926, Mrs N.E. Parry 7, K (K000820535!).

#### Lectotypification.

The protologue by Fischer indicates the specimen that was studied for the description of the species as “India. Assam, South Lushai Hills at Sairep, 1700 m. July, *Mrs N E.Parry 7*”. During our study we located five different sheets at Kew herbarium having the same collection number and locality as quoted above. Hilliard and Burtt (1995) noted that Parry’s numbers do not refer to individual collections, but instead they refer to unique species that she had recognized in the field. Weber et al. (2000) recognized three of these specimens as type material, but failed to designate a lectotype. One of these specimens, with a barcode number K000820535, has the collector’s original label which mentions ‘July 1926’ as the collection date. The author’s note on the label matches the note that Fischer has quoted in the protologue: “grows on rocky cliffs, leaves pale-green, silvered when dry, calyx light yellow, corolla orange red”. Since this is the only specimen where the collection number, month, and the author’s note matches the protologue, we designate K000820535 as the lectotype here.

#### Revised description.

Terrestrial or epilithic herbs, up to 20 cm tall, total height including inflorescence ca. 25 cm. Rhizome 1–2 × 0.5–1.0 cm. Stem 3–10 × 3.5–8 mm, erect, dark brown to light green, terete, pubescent with eglandular hairs, interspersed with yellowish cruciform pigment glands (in dried specimen). Leaves 1 – 4 pairs, opposite, anisophyllous, decussate, arrangement tufted in close pairs, terminal pair is reduced, exstipulate; petioles 4–9 cm long, light brown to light green, pubescent as on stem, interspersed with yellowish cruciform pigment glands (in dried specimen); lamina 6–12 × 5–10 cm, orbicular to ovate, base cordate to obliquely cordate, apex acute to subobtuse, margins crenate to serrate, dorsal surface dark green, pubescent with eglandular hairs, ventral surface light green, veins pubescent and intervals sparsely pilose, hairs eglandular, abaxial surface is covered with yellow to brownish cruciform pigment glands (in dry specimen); midrib with 6–8 secondary veins in either side, basal 3–4 pairs palmate, sunken above, raised below. Inflorescence 1–4, pedunculate, erect, axillary, pair-flowered dichasial cymes, arising from the axils of the 1–2 uppermost pairs of leaves, cyme with 12–16 flowers; primary bracteoles present, 8 × 4 mm, greenish-yellow, opposite, ovate, apex subacute, glabrous, abaxial surface covered with small cruciform pigment glands, secondary bracteoles yellow, present at the base of each cyme unit, 6 × 3 mm, thick, veins visible in dried specimens; orange flowers contrast against yellow calyces; peduncle 10–25 cm long, up to 2 mm wide, brownish at the base, light green towards apex, pubescent with eglandular hairs, sparsely covered with minute cruciform pigment glands as on bracteoles; pedicel 4–12 mm long, greenish-yellow, glabrous, pigment glands absent. Calyx up to 1 cm long, bright yellow, linear lanceolate, apex acute, lobes 5, free to base, conspicuously veined, glabrous, thick, persistent. Corolla 1.3–2.2 cm long, ca. 1.5 mm wide, tubular, tip infundibuliform, glabrous, corolla tube dorsally orange while ventral section of the corolla tube with a light yellow stripe running along the length of the tube, corolla lobes bilabiate, orange, glabrous, orbicular, upper lobes 2, 1.5 × 2 mm; lower lobes 3, 4 × 3 mm, spreading at right angles to the upper lobes, central lobe wider than the 2 lateral lobes. Stamens 2, oblong, glabrous, inserted near the throat of the corolla tube, anthers dorsifixed, coherent by adaxial surfaces; filaments 4–5 mm long, yellowish orange, glabrous; staminodes 2, 2 mm long, linear. Disc up to 1.2 mm, tubular with undulating upper margin, yellowish, glabrous, persistent. Gynoecium ca. 2 cm, ovary greenish yellow, linear, glabrous; style ca. 0.5 cm, glabrous; stigma capitate, green. Capsules 17–24 mm, linear, glabrous, orthocarpic. Seeds minute, ellipsoid, glabrous, muricate.

#### Amendments to protologue.

Upon examining the historical specimens and our fresh collections (Fig. 4) we believe that the indumentum on the stem is better described as pubescent rather than as strigose. Similarly, we describe corolla lobe shape as orbicular and not suborbicular (as mentioned in the protologue).

#### Note.

Differs from *D. tristis* Craib in having larger, bright yellow colored bracteoles and sepals respectively (maroon bracteoles and sepals in *D. tristis*). In addition, *D. parryorum* has orbicular to ovate leaves (oblong to lanceolate in *D. tristis*) and smaller corolla (1.3–2.2 cm in *D. parryorum*, 2–2.4 cm in *D. tristis*).

#### Distribution and vernacular name.

Historically, *D. parryorum* is known from only two districts (Lunglei and Lawngtlai) of southern Mizoram. We could locate the species only from the type locality (Sairep village in Lunglei district, specimen numbers: VG2018MZ2522, VG2018MZ2528, VG2018MZ2529, VG2018MZ2546). In Sairep, the plant is known as ‘*Chhakzhau*’ in local Mizo language.

#### Habitat.

This plant is generally found growing on moist loamy banks in partially shaded tropical wet evergreen forests.

#### Phenology.

Flowering in July to September, fruiting in August to December.

#### Conservation status and preliminary IUCN assessment.

*Didymocarpus parryorum* is historically known from only two localities in southern Mizoram, India: Lunglei and Lawngtlai districts. It has not been collected for the past 90 years, until this study in 2018, when we found it growing in its type locality. The extant population is restricted to a small patch of less than 10 km^2^ in a rapidly degrading forest and it has about 500 mature individuals. A village road passes through the plant’s habitat, further threatening its population. Therefore we propose that the species should be considered as critically endangered (CR) as per the B2ab criteria of IUCN guidelines (IUCN 2019).

### Didymocarpus wengeri

Taxon classificationPlantaeLamialesGesneriaceae

C.E.C.Fisch., Bull. Misc. Inform. Kew. 1928(2): 74. 1928.

531E8B1A-BED0-569B-A369-C3E120D2C195

**Figure 5.**
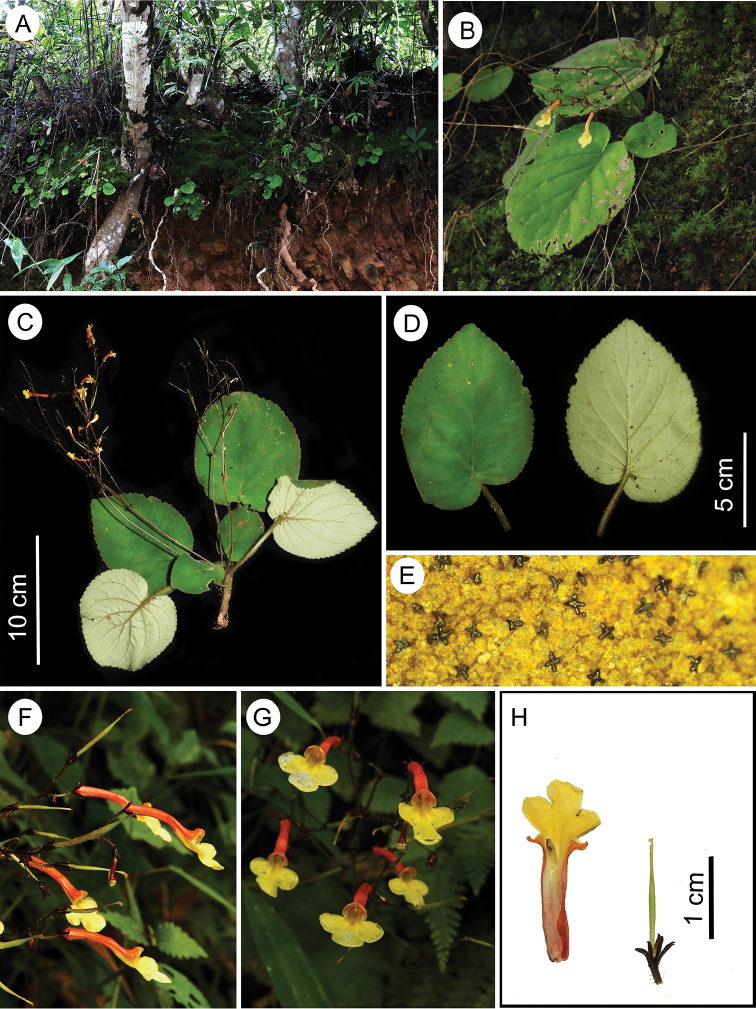
*Didymocarpus wengeri* C.E.C. Fisch. **A** habitat **B** habit **C** plant with inflorescence **D** leaf adaxial and abaxial surface **E** glands on the lower surface of leaves **F** flowers (lateral view) **G** flowers (frontal view) **H** floral dissection from left to right: opened floral tube, calyx with gynoecium. Photographs by NSP.

Fig. 5 , Suppl. material 1: Fig. S1F

#### Lectotype

**(designated here).** India, Assam (= Mizoram): South Lushai Hills, 2500 ft, comm. September 1927, Rev. W. J. L. Wenger 1, K (K000820530!).

#### Lectotypification.

There are only four historical collections of *D. wengeri*, two of which are specimens collected by Wenger, and the third by Parry. There is a fourth specimen at CAL, collected from southern Mizoram, which does not have the collectors’ details, but there is a possibility that it may be from Wenger or Parry’s collection, as the specimen was received from Kew herbaria and the script matches Fischer’s writing. In the protologue, Fischer indicated the specimen he studied for the description of the species as “Assam, South Lushai Hills, 2400 ft., Rev. W.J.L. Wenger’’, but he did not mention any specific collection date or number. We could not locate any specimen collected by Wenger at South Lushai hills at 2400 feet elevation in any herbaria (ARUN, ASSAM, CAL, E, K and BM), where Wenger’s specimens are known to exist. There is a specimen collected by Wenger (Wenger 1) at Kew (K000820530), without a collection date but with a note “*comm.* Sept 1927”, presumably written by Fischer. The label clearly mentions that the specimen was collected at ‘2500 ft.’ In their study, Weber et al. (2000) considered K000820530 as a type but they did not designate the status of the type: “Type: INDIA, Mizoram (previously Assam), South Lushai Hills, 2500 ft., IX. 1927. Wenger (K)”. We suggest that the ‘2400 ft.’ in Fischer’s protologue possibly is a typographical error, which has also been suggested by Weber et al. (2000), wherein the elevation has been cited as ‘2500 ft.’ by them and not as ‘2400 ft.’, as featured in the protologue. Given these observations, we designate K000820530 as the lectotype here.

#### Revised description.

Terrestrial or epilithic herbs, 7 cm tall, total height including inflorescence ca. 10 cm. Stem 5–60 × 3 mm, subacaulascent to 6 cm, terete, light green to dark maroon, villose with 4–10 celled glandular hairs (rarely eglandular), densely covered with cruciform pigment glands. Leaves 1–4 pairs, opposite and anisophyllous, decussate, terminal pair smaller in size, arrangement tufted in close pairs, exstipulate; petioles 2–5 cm long, villose with 4–10 celled eglandular hairs, glands cruciform, density and structure similar to the ones on stem; lamina 1.8–6 × 1.5–6 cm, orbicular, base cordate and often unequal, apex sub-obtuse, margin crenate to serrate with multicellular hairs; dorsal surface dark green, villous with eglandular hairs, ventral surface, densely villose along veins but sparsely villose otherwise; lower lamina covered with cruciform, dark brown pigment glands (in dried specimen), dense along the (midrib) and veins; midrib with 5–8 lateral veins on either side, basal 3–5 veins palmate, sunken above, raised below. Inflorescence 1 to 5, pedunculate, axillary, pair-flowered cymes usually arising only from the axils of the 1–2 uppermost pairs of leaves, cyme with 6–16 flowers; primary bracteoles present, 2 × 1 mm, opposite, lanceolate, reddish brown, sometimes with eglandular hairs, below densely covered with cruciform glands; secondary bracteoles (within the cyme) present at each dichotomous fork, 1–2 mm in diam., orbicular, reddish brown, sparsely covered with eglandular hairs, hirsute on upper surface, glandular hairs on lower surface; peduncle 5–15 cm long, dark maroon, primary axis is sparsely covered with both glandular and eglandular hairs, secondary axis with glandular hairs, cruciform pigment glands present near the base of the primary axis and at the fork; pedicel 2–10 mm long, glandular hairs present. Calyx 5 lobes, 1.5–3.5 mm long, free to base, linear lanceolate, glabrous, dark brown to maroon, persistent. Corolla 0.8–2.2 cm long, ca. 2–3 mm wide, tubular, tip infundibuliform, orange-red at the base and yellow ventrally from throat to mouth, yellow extending into the lower lobes, corolla bi-lipped, total 5 lobes, yellow, upper 2 lobes fused with 2 × 2 mm, orbicular; lower lobes 3, 7 × 4 mm, orbicular, spreading at right angles to the upper lobes, middle/central lobe wider than the 2 lateral lobes, glabrous. Stamens 2, inserted near the throat of the corolla tube; filaments 3–4 mm long, glabrous, filament yellowish, anthers oblong, dorsifixed, coherent by adaxial surfaces, pubescent; staminodes absent. Disc up to 1.5 mm, tubular with undulating upper margin, greenish yellow, glabrous, persistent. Gynoecium 10–12 mm, ovary greenish yellow, linear, indistinct from stipe, glabrous; style ca. 4 mm glabrous; stigma yellow, capitate. Capsule 1.5–2.5 cm long, linear, glabrous, dehiscence longitudinal. Seeds data deficient.

#### Amendments to protologue.

The protologue mentions that disc is absent at the base of the ovary. Fresh specimens show the presence of small tubular disc at the base of the ovary (Fig. 5 H).

#### Note.

This species is similar to *D. margaritae* W.W.Sm., but differs from it in having cruciform brownish glands on abaxial surface of the leaves (glands absent in *D. margaritae*) and has yellow colored corolla lobes (orange corolla lobes in *D. margaritae*). Peduncle with glandular hairs in *D. wengeri*, whereas peduncle of *D. margaritae* is glabrous.

#### Distribution.

All collections of *D. wengeri*, including the type specimen, are from southern Mizoram. In our study, we located a population from Tuipang in the Saiha district of southern Mizoram (specimen numbers: VG2018MZ2556, VG2018MZ2557, VG2018MZ2558). This locality corresponds to where Parry had collected specimens in 1928. In addition, we found a second population in the Lawngtlai district of southern Mizoram (specimen numbers: VG2018MZ2568, VG2018MZ2569, VG2018MZ2570).

#### Habitat.

Growing on steep clay banks along the roads in partially shaded, tropical wet evergreen forests.

#### Phenology.

Flowering in August to September, fruiting in October to January.

#### Conservation status and preliminary IUCN assessment.

*D. wengeri* is currently known from only two locations in southern Mizoram, India: Saiha district and Lawngtlai district. Only one population each has been located in these two districts and they are separated by a distance of 135 km. This rediscovery is after a span of 87 years and a total of 52 individuals were found during the flowering season of 2018. In the protologue, Fischer has quoted Wenger’s (collector) note as “apparently scarce, at least in these hills, for I have only found one small patch on a steep clayey bank”, indicating that these plants were very rare even when they were first collected. Considering the small, fragmented population and rapidly degrading habitat, the species should be considered as critically endangered (CR) as per C2a(i) of the IUCN guidelines (IUCN 2019).

## Discussion

Northeast India, and the state of Mizoram in particular, has been floristically underexplored, and our study highlights the need for more careful and extensive biodiversity studies in this region. The rediscovery of three species close to their type localities and the collection of one away from its type locality (*D. adenocarpus*) suggests that conservation of the narrow endemics is critical and understanding their biology should be prioritized. The geographic placement of Mizoram (between Bangladesh and Myanmar) also indicates that cross-border biogeographic studies should be carried out to understand the dispersal and evolution of plants in this region.

## Supplementary Material

XML Treatment for Didymocarpus adenocarpus

XML Treatment for Didymocarpus lineicapsa

XML Treatment for Didymocarpus parryorum

XML Treatment for Didymocarpus wengeri

## Appendix 1

**List of *Didymocarpus* specimens examined**

***Didymocarpus adenocarpus* C.E.C.Fisch**.: INDIA, Mizoram. S. Lushai hills, September 1928, Rev. W.J.L. Wenger 239, K (K000820546); S. Lushai hills, September 1931, Rev. W.J.L. Wenger 347, K (K000820547); S.Lushai hills, September 1931, Rev. W.J.L. Wenger 347, E (E00627876); Sairep, September 1927, Mrs N.E. Parry, No.251, K; Reiek Tlang, 25 August 2018, Prasanna N.S., VG2018MZ2589, BHPL; Reiek Tlang, 25 August 2018, Prasanna N.S., VG2018MZ2590, BHPL; Reiek Tlang, 25 August 2018, Prasanna N.S., VG2018MZ2592, BHPL.

***Didymocarpus purpureobracteatus* W.W.Sm.**: CHINA, Yunnan. S. of Red River, without date, Henry 9746A, US (US00056528); San Teo Chen, without date, A. Henry 9189, NYBG (NY00063234).

***Didymocarpus lineicapsa* (C.E.C.Fisch.) B.L.Burtt**: INDIA. Mizoram: S. Lushai, August 1928, Rev. W.J.L. Wenger, No.238, E (E00627917); Aizawl, September 1927, Mrs N.E. Parry, No.79, K (K000820539); Aizawl, March 1928, Mrs N.E. Parry, No.79, K (K000820540); Aizawl, 1928, Mrs N.E. Parry, No.79. K (K000820541); S. Lushai, August 1928, Rev. W.J.L. Wenger, No.238 K; Hmuifang, August 1927, Mrs N.E. Parry, No.262, K; Hmuifang, August 1927, Mrs N.E. Parry, No.259, K; Mamit, Reiek Village, August 2018, Prasanna N.S., VG2018MZ2581, BHPL; Mamit, Reiek Tlang, August 2018, Prasanna N.S., VG2018MZ2584, BHPL; Mamit, Reiek Tlang, August 2018, Prasanna N.S., VG2018MZ2585, BHPL; Mamit, Luanpawl to Dawpui road, August 2018, Prasanna N.S.,VG2018MZ2496, BHPL.

***Didymocarpus graciliflorus* R.W.MacGregor & W.W.Sm.**: MYANMAR. S. Shan States: Keng Tung, August 1909, MacGregor, R.W., No.714, K, (K000820542).

***Didymocarpus parryorum* C.E.C.Fisch.**: INDIA. Mizoram. Lunglei, Sairep, July 1926, Mrs N.E. Parry No.7, K (K000820535); Sairep, 1928, Mrs N.E. Parry No.7, K, (K000820536); Sairep, December 1927, Mrs N.E. Parry No.7, K, (K000820537); Phongpui, March 1927, Mrs N.E. Parry No.NA (2 sheets) K; N.Vanlaphai,’comm’ January 1928, Mrs N.E. Parry CAL (CAL0000019176); Sairep, August 2018, Prasanna N.S., VG2018MZ2522, BHPL; Sairep, August 2018, Prasanna N.S., VG2018MZ2528, BHPL; Sairep, August 2018, Prasanna N.S., VG2018MZ2529, BHPL; Sairep, August 2018, Prasanna N.S., VG2018MZ2546, BHPL.

***Didymocarpus tristis* Craib**: THAILAND, Chanthaburi, Khao Khitchakut, 27 Aug 2012, Middleton et al, 5673, E.

***Didymocarpus wengeri* C.E.C.Fisch.**: INDIA. Mizoram. S. Lushai, August 1931, Rev. W.J.L. Wenger (collectors number not mentioned), E, (E00627988); South Lushai hills, (without date, comm. September 1927), Rev. W.J.L. Wenger, 1, K, (K000820530); Lushai hills, Tuipang, January 1928, Mrs N.E. Parry, 452, K, (K000820531); S. Lushai, without collectors name, and date, CAL (CAL0000019172); Saiha, August 2018, Prasanna N.S., VG2018MZ2556, BHPL; Saiha, August 2018, Prasanna N.S., VG2018MZ2557, BHPL; Saiha, August 2018, Prasanna N.S., VG2018MZ2558, BHPL; Lawngtlai, August 2018, Prasanna N.S., VG2018MZ2568, BHPL; Lawngtlai, August 2018, Prasanna N.S., VG2018MZ2569, BHPL; Lawngtlai, August 2018, Prasanna N.S., VG2018MZ2570, BHPL.

***Didymocarpus margaritae* W.W.Sm.**: CHINA, Yunnan. Szemoa. Henry, A., #12380b, US, (US00056517).

## Supplementary materials

Supplementary material 1 Figure S1 Data type Multimedia Explanation note **A** Underside of *D. adenocarpus* C.E.C.Fisch. plant showing inflorescence hidden under leaves **B** Watery calyx of *D. adenocarpus* C.E.C.Fisch. **C** Lateral view of *D. parryorum* C.E.C.Fisch. flower showing color stripe along the floral tube **D** Tridentate calyx lobe of *D. lineicapsa* (C.E.C.Fisch.) B.L.Burtt **E** Inflorescence of *D. parryorum* C.E.C.Fisch. **F** Inflorescence of *D. wengeri* C.E.C.Fisch. Photographs by NSP. https://binary.pensoft.net/file/412035This dataset is made available under the Open Database License (http://opendatacommons.org/licenses/odbl/1.0/). The Open Database License (ODbL) is a license agreement intended to allow users to freely share, modify, and use this Dataset while maintaining this same freedom for others, provided that the original source and author(s) are credited.Naibi Shrungeshwara Prasanna, Vinita Gowda
