# Supplementary figures and images for: Rediscovery of four narrow endemic Didymocarpus species (Gesneriaceae) from Mizoram, India, with revised species descriptions and lectotypifications
Source: PhytoKeys. 2020 May 20;148:1–19. doi: 10.3897/phytokeys.148.49772 (PMC7253502; doi:10.3897/phytokeys.148.49772)

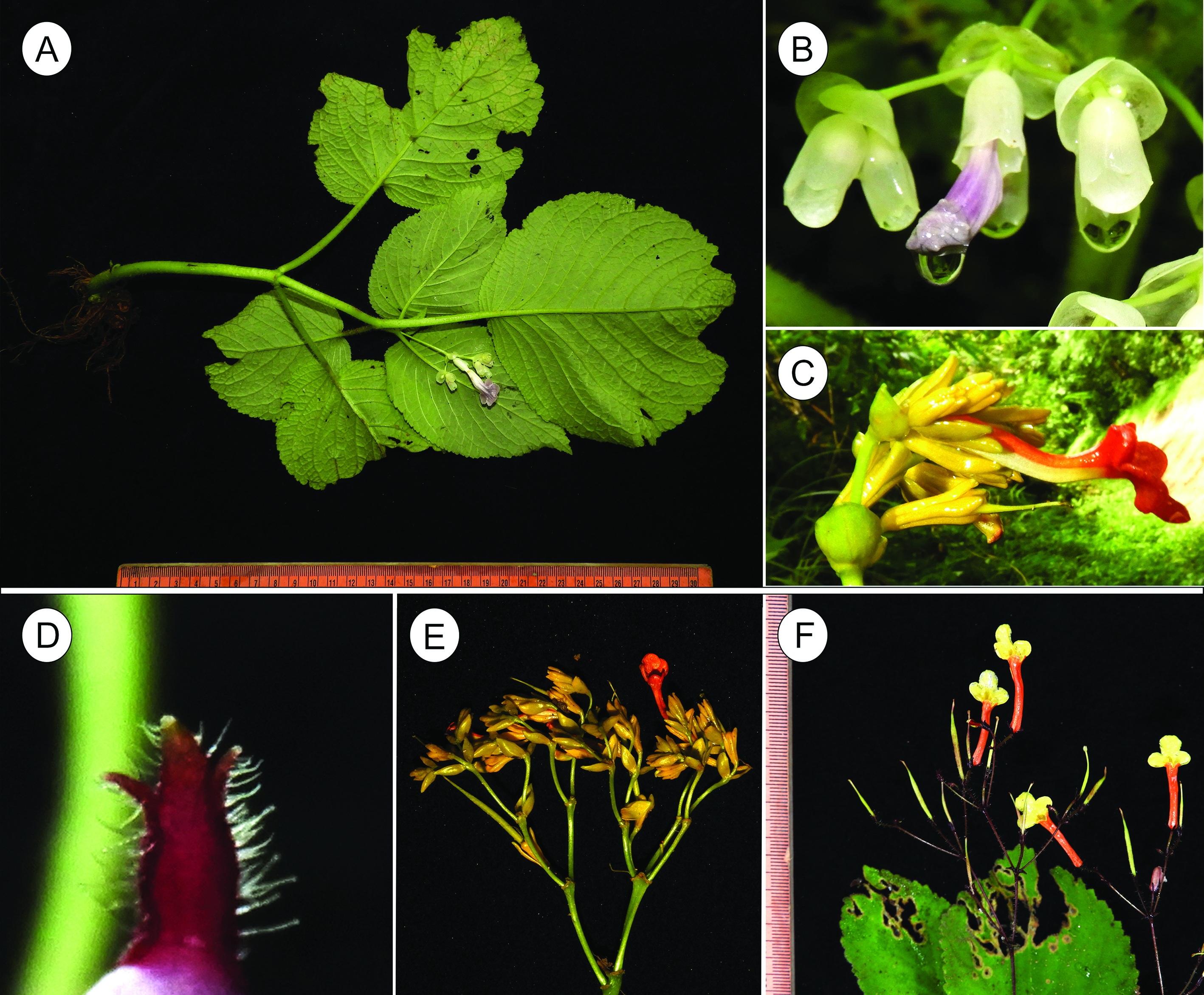

Supplement: Supplementary material 1 — Figure S1 [file phytokeys-148-001-s001.tif]
